# Shell game: Neanderthal use of the European pond turtle (*Emys orbicularis*) in the Last Interglacial landscape of Neumark-Nord (Germany)

**DOI:** 10.1038/s41598-026-42113-x

**Published:** 2026-04-08

**Authors:** Sabine Gaudzinski-Windheuser, Svenja Böll, Antje Griesch, Lutz Kindler, Wil Roebroeks

**Affiliations:** 1 MONREPOS Archaeological Research Centre and Museum for Human Behavioural Evolution (LEIZA), Schloss Monrepos, 56567 Neuwied, Germany; 2 Institute of Ancient Studies, Pre- and Protohistoric Archaeology, Johannes Gutenberg-University Mainz, Hegelstraße 59, 55122 Mainz, Germany; 3 Landeskriminalamt Nordrhein-Westfalen, Völklinger Straße 49, 40221 Düsseldorf, Germany; 4 Faculty of Archaeology, Leiden University, P.O. Box 9514, Leiden, 2300 RA The Netherlands

**Keywords:** Neanderthals, Last Interglacial, Prey diversity, Subsistence, Small game, Pond terrapin, Ecology, Ecology, Evolution, Zoology

## Abstract

Data on palaeolithic subsistence is often obtained through studies of faunal palimpsests, containing remains of animal processing activities accumulated over non-quantifiable amounts of time. Compounding such site-specific data with evidence from other sites distributed over large areas - i.e. integrating data spanning large temporal as well as spatial scales - results in coarse-grained reconstructions of past prey diversity. In contrast, here we present prey diversity data from what is—geologically speaking—a “snapshot” of a ~ 25-hectare area frequented by Neanderthals during the Last Interglacial, with a focus on their exploitation of the pond terrapin *Emys orbicularis*. These data constitute the first evidence of turtle exploitation by Neanderthals north of the European mountain chains, beyond the Mediterranean basin. This Neumark-Nord record demonstrates that Last Interglacial foragers exploited a wide range of archaeologically visible resources available in this lake area, from small (~ 1 kg) pond terrapins up to and including the largest terrestrial mammals of the Pleistocene, straight-tusked elephants, with adult males weighing more than 10 tonnes. The abundance of intensively exploited medium- and large-sized mammals found alongside these *Emys* remains suggests that other variables than macronutrients per se played a role in the repeated harvesting of pond terrapins from these water bodies.

## Introduction

In recent years, the diversity of Neanderthal prey choice has become well established, underlining their ecological adaptability and flexibility as well as a strong overlap with subsistence activities of Upper Palaeolithic *Homo sapiens*. Beyond traditionally recognized medium- to large-sized mammals—such as horses, bovids, and deer— a broad spectrum of smaller mammals - including leporids^1^, birds and reptiles^2^ has been added to the Neanderthal dietary repertoire, with straight-tusked elephants with a weight up to 135 tonnes on the other end of their prey size distribution^2–5^. Additionally, evidence for the consumption of freshwater and marine resources, including shellfish and crabs^6,7^, has been documented across the Mediterranean basin and the southwest Iberian peninsula. Together with data on plant consumption^8,9^, with evidence for large-scale grease rendering^10^ and the suggestion of maggot ingestion as a potentially important dietary component^11^, a more nuanced and more complex picture of the dietary breadth and nutrition of Middle and Late Pleistocene Neanderthals has emerged.

Tortoises and turtles, members of the order Testudines, are amongst the smaller prey animals exploited by Neanderthals, well-documented throughout the circum-Mediterranean region, particularly in Iberia^12–14^, Italy^15^, and the Levant^16^. In some cases, e.g., at Kebara^16^, tortoise exploitation may have been sufficiently intensive to have led to reductions in average body size. For the Portuguese site of Gruta da Oliveira, it has been suggested that intensive exploitation of *Testudo hermanni* during Marine Isotope Stage (MIS) 5 contributed to a localized extinction of the species at the onset of MIS 4^13^.

To date, evidence for Neanderthal exploitation of testudines is confined to the wider Mediterranean basin, which thus far also yields the bulk of the data on small prey use by Neanderthals. This southern distribution is consistent with the ecological requirements of tortoises and turtles, which depend on warm summer temperatures for successful egg incubation. The land-dwelling Hermann’s Tortoise is a Mediterranean form with a marked thermophilic character, while the modern distribution of the European pond turtle (*Emys orbicularis*) in western and central Europe is restricted to regions above the 20 °C July isotherm. This inferred strong (climatic) link between the latter species’ present northern and eastern range limit and specific July temperatures is somewhat debatable though, given a long history of intense human predation which has led to its disappearance from numerous areas within Europe^17,18^. Historically, *E. orbicularis* was widely consumed in areas where it occurred, not only as a food source but also for medicinal purposes, until it became increasingly rare in the 19th century^19^.

In the Iberian peninsula, *T. hermanni* is the most common tortoise species represented in the archaeological record^13^. With an adult carapace length ranging from 15 to 28 cm, *T. hermanni* is generally larger than *E. orbicularis*, which typically measures 12–23 cm in carapace length, its smaller size and lighter build an adaptation to its aquatic lifestyle. While rare in the Iberian record, a recent study has established the presence of *E. orbicularis* in Middle Palaeolithic levels at Gruta Nova da Columbeira in central Portugal^14^.

North of the major mountain chains of the Alps and Pyrenees, *E. orbicularis* appears in the Pleistocene fossil record only during the warm-temperate interglacial periods, which represent a relatively short (~ 10–15%) span of Middle and Late Pleistocene time. With the post-glacial warming of the Holocene, *Emys* rapidly expanded its range from southern European refugia, reaching as far north as southern Sweden by approximately 9,800 years ago^17^ and appearing in archaeological contexts across western and central Europe from the Mesolithic onward.

Syntheses of Neanderthal prey diversity typically integrate data spanning large temporal as well as spatial scales to obtain comprehensive overviews. Data on former subsistence are usually obtained through studies of faunal palimpsests, containing the remains of animal processing activities accumulated over non-quantifiable amounts of time. Compounding such site-specific data with evidence from other sites, often distributed over large areas - e.g. the “circum-Mediterranean” -, creates a coarse-grained picture of prey diversity in the deep past. In contrast to the mostly rock-shelters based evidence mentioned above, the data presented here derives from the localized (~ 25 ha) open-air and high-sedimentation rate context of the Neumark-Nord (Germany) Last Interglacial lake landscape: it covers a geologically speaking very short time span, during which high-resolution data attest to Neanderthal exploitation of a remarkably diverse array of prey species—including the largest terrestrial mammals of the Pleistocene, straight-tusked elephants (*Palaeoloxodon antiquus*)^20^.

### Neumark-Nord

**Fig. 1:**
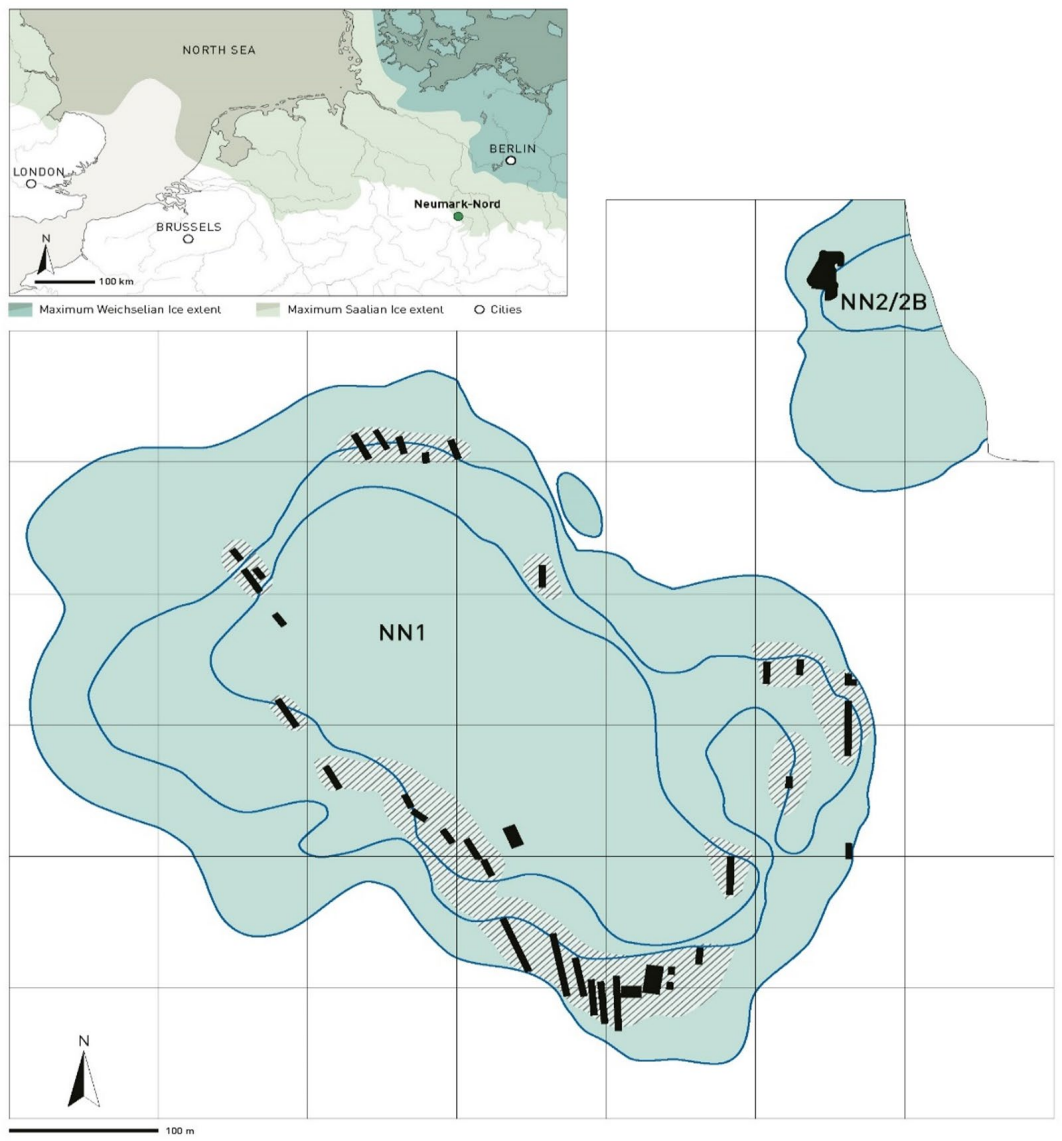
Outline of Basin NN1 and NN2 in the former lignite mine Mücheln (Geiseltal). In black the locations of short rescue interventions in basin NN1, and of the multi-year NN2/2B excavation in the small basin NN2 (scale bar 100 m). The inset shows the geographic location of Neumark-Nord on the northern European plain, relative to the maximum extension of the last (Weichselian) and penultimate (Saalian) glaciers.

The Last Interglacial basins of Neumark-Nord were exposed in long-term archaeological and geological fieldwork in the former lignite open-cast mine Mücheln in Saxony-Anhalt, Germany^21–23^(Fig. 1). Basin Neumark-Nord 1 (NN1), uncovered in 1985, was investigated thoroughly by Dietrich Mania and his research group in a series of rescue interventions, in a continuous race against the large-scale bucket-wheel excavators, until the end of the mining activities in the mid-1990s^21^. During subsequent reclamation works in the quarry, Mania found a second basin, Neumark-Nord 2, about 100 m northeast of Neumark-Nord 1^23^. Multidisciplinary investigations and archaeological excavations of basin Neumark-Nord 2 were undertaken from 2004 to 2009. Lake basin Neumark-Nord 1 covered an area of about 24 ha, while basin Neumark-Nord 2 (NN2) represents a small and shallow pond, of about 1.6 ha in documented size. Neanderthals left abundant traces of their presence on the shores of these two water bodies^10,20,21,24,25^, whose synchronous interglacial infill and detailed environmental records provide a solid temporal framework to track Neanderthal activities within an interglacial landscape (Fig. 1). Their archaeological record dates to the first 7000 years of the Eemian interglacial, with most archaeology dating to the early Eemian, to Pollen Assemblage Zones (PAZ) III and IV, with a total duration of ~ 2850 years^26^. This record includes high-density flint and bone scatters, such as excavated in great detail at Neumark-Nord 2/2B^10^, as well as low-density ones, including single-skeleton kill and butchering locations.

During periods of regression of the large Neumark-Nord 1 lake, up to 100 m broad littoral zones developed in the basin, with the so-called *Untere Uferzone* (Lower Shore Zone) and the *Obere Uferzone* (Upper Shore Zone) especially well-documented. In this Unit 6 complex, high-density find concentrations were encountered, which could usually not be excavated properly because of active mining there. These concentrations were characterized by the presence of highly fragmented animal bones, middle palaeolithic flint artifacts - knapping waste, unmodified flakes from mostly simple discoidal cores, some scrapers and denticulates - and charcoal particles^21^. In addition, especially in the Lower Shore Zone, along the immediate shore, clusters of disarticulated and partly articulated herbivore carcasses were uncovered by the rotary extractors. In some instances, the presence of simple flakes as well as cut marks suggests Neanderthal involvement in the disarticulation of the carcasses. Estimates of sedimentation rates, development of peat layers and counting of annual rings in tree stumps indicate that the Lower Shore Zone (Unit 6.1) at Neumark-Nord 1 existed for c. 300 years^21,22^. The majority of the *Emys* material published here (NISP 67) derives from this Neumark-Nord 1 Lower Shore Zone, as does most of the *P. antiquus* remains mentioned above.

**Fig. 2:**
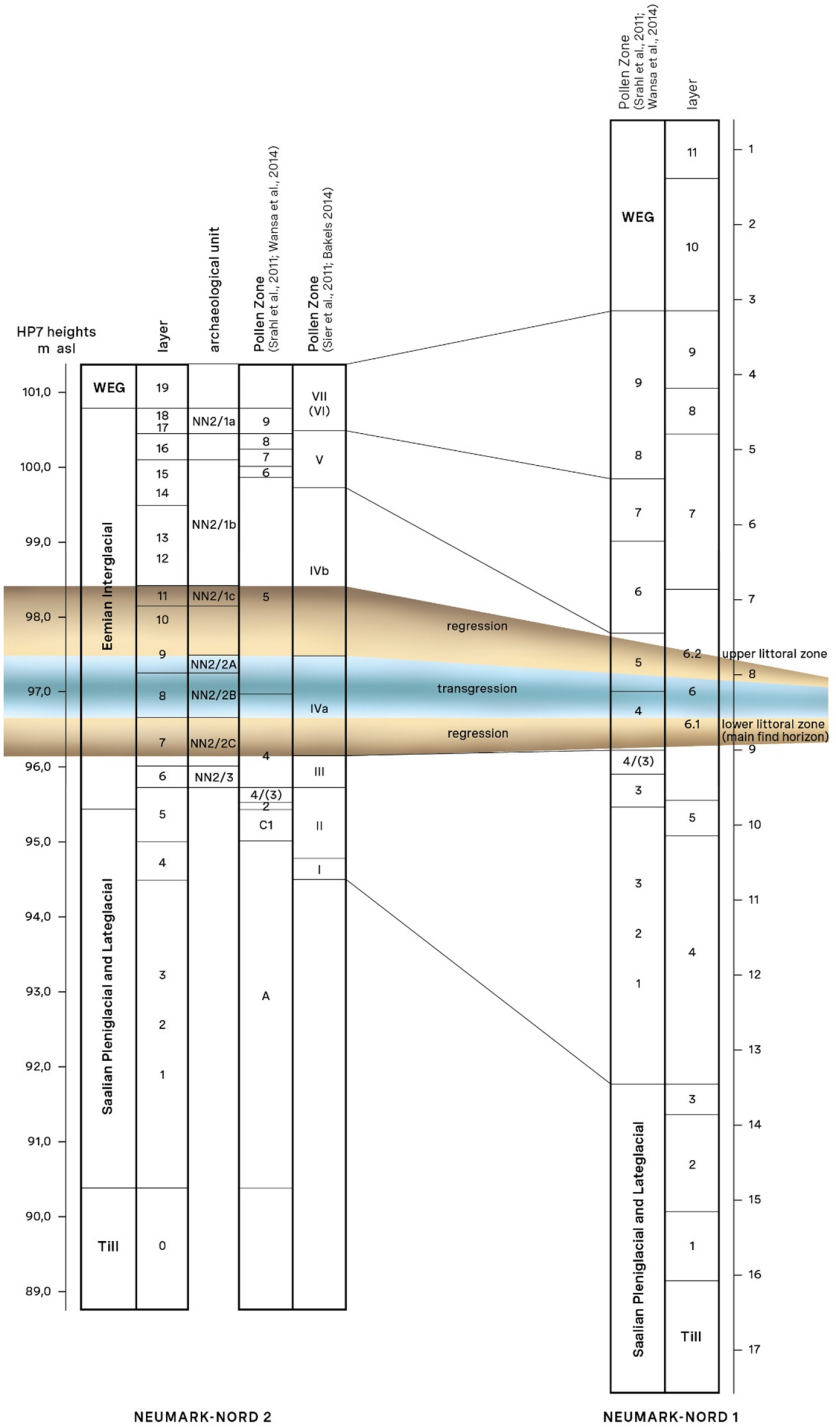
Stratigraphic correlation of the NN1 and NN2 basin sequences, with the Late Saalian till at the base and the Weichselian Early Glacial (WEG) deposits covering the basins infill. In colour indicated the deposits related to major transgression and regression phases from which the *Emys orbicularis* remains derive. Pollen zones for the Eemian follow the subdivisions established by respectively Erd^29^ (PAZ 1–9) and by Menke and Tynni^26^ (PAZ I-VII). Unit 6.1 in the NN1 profile = Lower Shore Horizon, Unit 6.2 in NN1 profile = Upper Shore Horizon. (Profile updated from^30^.

The systematic and long-term excavations at the Neumark-Nord 2 basin^21–23^ likewise yielded remains of the pond terrapin (NISP = 25), associated with the Neumark-Nord 2/2B grease rendering location^10,23^, developed during the lake transgression phase that separates both littoral zones in layer 6 of Neumark-Nord 1 (Fig. 2). Together with the Lower Shore Horizon, the find bearing unit 8 at Neumark-Nord 2/2B can be assigned to the *Corylus* zone of the Eemian (PAZ IVa)^27^, with an estimated duration of ~ 1200 years. Within that *Corylus* phase, the Neumark-Nord 2/2B material accumulated in maximally 288–455 years^10^. The Neumark-Nord 2/2B *Emys* material constitutes a very small part of the 118,744 faunal remains recovered at that site in a remarkable concentration, containing the remains of minimally 172 intensively exploited large mammals associated with 16,524 flint artifacts^10^.

The stratigraphic position of all *E. orbicularis* specimens studied here covers a time interval of approximately 900 years during PAZ IV^24^, which is characterized by a virtually continuous presence of Neanderthals along the Neumark lakes, associated with evidence for anthropogenic fire-related landscape modification^28^. From a zooarchaeological perspective, Neanderthal subsistence activities during this period included intense predation of megaherbivores - such as elephants^20^– and medium and large sized herbivores at the lake margins and carcass accumulations in the lake land for grease rendering^10^(Table 1).

**Table 1:** Overview of the mammal taxa recovered from the Neumark-Nord 1 and 2 sedimentary units shown in Fig. 2. Numbers indicate Minimum Number of Individuals (MNI) identified (updated from^30^; see also^10^.

Archaeological layer	1 st regression phase	transgression phase		2nd regression phase	
	NN1.6.1 lower littoral zone	NN1.6 silty and coarse sandy gyttja	NN2/2B	NN1.6.2 upper littoral zone	NN2/1c
Taxon/MNI					
*Panthera leo/* cf. *Panthera leo*	2	1	2		
*Crocuta crocuta*	1			1	1
*Ursus* sp.	1		8		
*Canis lupus*	1		1		
*Vulpes vulpes*			1		
*Palaeoloxodon antiquus*	52	9	2		1
Rhinocerotidae	9	2	2	2	
*Sus scrofa*			1		
*Equus* sp.	3		56		2
*Bos primigenius*	8	2	45	1	2
smaller Cervids (*Capreolus*-*Dama-Cervus* size)	3	26	20		2
larger Cervids (*Cervus*–A*lces*-*Megaloceros* size)	4	6	34		3
Sum	84	46	172	5	11
	318				

## Results

Most of the *E. orbicularis* remains from Neumark-Nord 1 were recovered by Dietrich Mania and the geologist Matthias Thomae, as a by-product of their fieldwork surveying and recording the Pleistocene sections created by the mining activities in the huge lignite quarry. All remains were isolated pieces, with these finds constituting the smallest size fraction of the find material recovered from the unit 6 deposits there. As reported by Böhme^31^, subsequent dedicated surveys of the shore deposit sections by Böhme and Heinrich yielded additional material, again all finds retrieved from the unit 6 deposits, almost all from the Lower Shore (*Untere Ufer*) horizon (6.1) part of that unit.

We recorded a total of 92 shell fragments of *E. orbicularis.* Within the Neumark-Nord 1 collection 67 fragments were identified, representing a slight increase from the initial 55 specimens described by Karl^32^ and the 62 fragments documented by Böhme^31^. All *Emys* finds reported by Böhme consist of fragments of carapace and plastron, with very few pieces displaying some signs of rounding which occurred in the shore area, but most being very well-preserved. Böhme additionally mentioned a left distal humerus fragment from unit 6.2. (the Upper Shore horizon), that was not available for the present study.

**Fig. 3:**
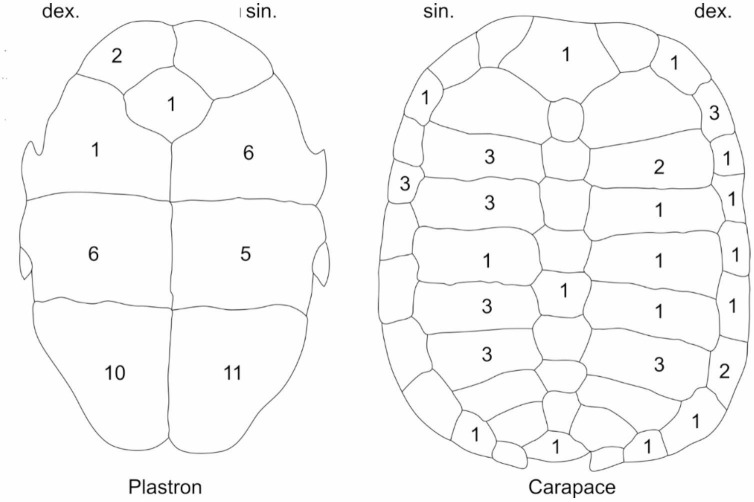
Number of identified specimens (NISP) of *Emys orbicularis* from Neumark-Nord. From the 92 bones of the European pond turtle 83 (= 90%) could be determined to a specific element and side of the plastron and carapace.

Of the 67 fragments studied, 27 consisted of carapace fragments, 40 deriving from the plastron (see Fig. 3; Table 2). That more fragments of the plastron were found than of the carapace could result from plastron fragments being larger and thicker, less prone to transport and easier to detect when surveying sections in an active quarry situation (the carapace consists of 49 individual thin parts, whereas the plastron is made up of 9 larger and thicker fragments. Only two parts of the plastron could be conjoined together and assigned to one individual. The remaining fragments of carapace and plastron do not fit together due to their different sizes and individual suture differences, so that each element must be assigned to an individual, according to Böhme^31^. His study of the NN1 sample concluded that it contained the remains of minimally 30 individuals, with reconstructed carapace lengths between 11 cm and 17 cm. Böhme suggested that most of the individuals were males, based on the morphology of the plastron^31^.

In the studied sample from Neumark-Nord 1, 41 finds derive from the Lower Shore, 7 from the Upper Shore and 14 specimens from the lake deposits separating both shorelines. Five specimens lack information on the depositional environment in sedimentary unit 6. From the NISP (number of identified specimens) of the individual bone elements present in each sub-unit an MNI (Minimum Number of Individuals) of 7 for the Lower shore, an MNI of 2 for the Upper Shore and an MNI of 3 for the lake deposit can be established.

In addition, the Neumark-Nord 2 excavation yielded four elements of the plastron and 20 elements of the carapace, as well as one shell fragment that could not be identified further. The specimens represent an MNI of 4 (see Fig. 3; Table 2).

**Table 2:** Number of identified specimens (NISP) of *Emys orbicularis* from sedimentary unit 6 in lake basin NN1 and from layer NN2/2B in NN2.

NISP	NN1			NN2		
	sin.	indet.	dex.	sin.	indet.	dex.
Carapace						
Nucal					1	
Neural I-VIII			2			
Metaneural			1			
Pleural I-VIII	10		7	3	4	1
Peripheral I-XI	2		5	3	2	6
**Plastron**						
Epiplastron			2			
Entoplastron					1	
Hyoplastron	6		1		1	
Hypoplastron	5		6			
Xiphiplastron	11		9			1
Car./Plast. indet.					2	
SUM	67			25		

Adult *E. orbicularis* individuals exhibit pronounced sexual dimorphism in plastron curvature, with females displaying flat to convex hyoplastron, hypoplastron, and xiphiplastron plates, while males show a more concave curvature. Based on these morphological characteristics, 19 plastron remains could be assigned to sex: 7 female and 12 male individuals, giving a male to female ratio of 1:0.58.

Carapace length in adult *E. orbicularis* ranges from 15 to18 cm, occasionally reaching up to 23 cm^33^. Northern and eastern subspecies generally attain larger sizes than southern populations^34^, with females typically exceeding males in size. Size estimation was performed using a conversion factor applied to hypoplastron width measurements (hypoplastron width × 3.5), derived from modern comparative material^31^. Calculated carapace lengths ranged from 10.9 to 16.5 cm. These values are comparable to Last Interglacial populations from Schönfeld, Germany^35^, but smaller than Holocene specimens from Denmark, which reached up to 19 cm^36^. Two specimens with carapace lengths under 12 cm were classified as juvenile^31^.

**Fig. 4:**
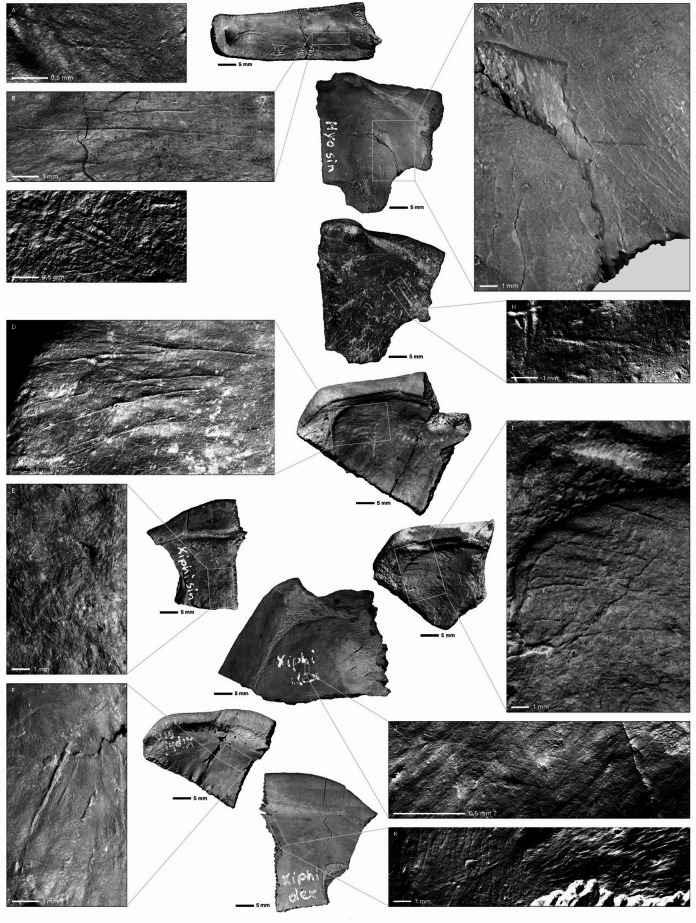
Cut marks on *E. orbicularis* remains from Neumark-Nord 1 (with find numbers in caption). Cut marks on fragments of the Carapace: **A**. Pleurale V dex., 87:300, 226/228; **B**. Pleurale IV dex., NN87:300, 708; **C**. Pleurale VI dex., no Findnumber. Cut marks on fragments of the Plastron: **D**. Xiphiplastron sin., 87:300, 314; **E**. Xiphiplastron sin., 87:300, 696; **F**. Xiphiplastron sin., 87:300, 18/20; **G**. Hyoplastron sin., 87:300, 175; **H**. Hyoplastron sin., 16-06-94; I. Xiphiplastron dex., 92:909, NN38; **J**. Xiphiplastron dex., 87:300, 652; **K**. Xiphiplastron dex., 87:300, 242. Images obtained through high resolution 3D-scans (LIM ToolScan R360).

Cut marks (Fig. 4) were identified on 22 fragments (Neumark-Nord 1 = 19, Neumark-Nord 2/2B = 3), all on the interior side of the bones. Most of the Neumark-Nord 1 cut marks occurred on elements of the plastron (15 of 40 plastron fragments, i.e. 37.5%), with only 4 of to the 27 carapace elements (i.e. 6.75%) displaying cut marks. Cut-marked elements include 4 carapace pleurals (pleural III sinister, pleural IV dexter, 2 pleural VI elements) and 15 plastron components (3 hyoplastra, 3 hypoplastra, 7 xiphiplastra, 1 epiplastron). A total of 70 cut marks were documented on the 15 plastron remains, while four carapace fragments displayed in total 16 almost exclusively parallel and short cut marks, in the direction of the vertebral column. Cut mark lengths range from 0.4 to 9.8 mm (mean: 3.1 mm), with no substantial difference between carapace (3.0 mm) and plastron (3.5 mm) elements. The number of cut marks per element varies from 1 to 9, averaging 4.1 for carapace and 4.6 for plastron fragments. Cut mark depth consistently measures less than 0.5 mm. Cut marks on the four pleural elements are located precisely at shoulder and pelvic girdle attachment sites, indicating removal of skeletal elements including clavicle and pelvis, along with limb disarticulation. Fine cut marks along bone margins suggest removal of soft tissues and possibly internal organs. The careful cleaning of carapace elements suggests their preparation for secondary use, potentially as containers.

Plastron cut marks show a less uniform distribution. While some cuts can be attributed to skeletal element removal and limb disarticulation, the lack of fusion between plastron and skeleton in this species suggests primary focus on soft tissue and organ removal. Three xiphiplastra display parallel cut marks extending from centre to margin, consistent with meat and organ extraction. Similar patterns have been documented by Blasco^12^ and attributed to soft tissue processing.

In the Neumark-Nord 2 material cut marks were observed on three elements of the carapace. Two pleural elements display on the inner side three parallel cut marks each, one additional cut mark is located on the inner side of a peripheral element. The pleural bones belong to the section close to the shoulder and pelvic gridle, the cut marks represent disarticulation of these elements. The cut mark on the peripheral bone may stem from the initial stage of butchery, during opening and separation of the plastron.

## Discussion

European pond turtles inhabit aquatic environments and rapidly submerge, hiding in water, silt or vegetation when disturbed, thereby probably presenting somewhat greater capture challenges than land dwelling tortoises such as *T. hermanni*. Nevertheless, compared to other prey animals, pond turtles are relatively easy to capture when encountered, with low processing costs, for example simply placing them over hot coals. However, while some have emphasized the nutritional value of testudines, the amount of protein and fat that can be derived from a one-kilogram *E. orbicularis* is very low: the reason why it has been suggested that the animal may have served as a food supplement^37^ at best. Given the abundant zooarchaeological evidence for intensive exploitation of large amounts of medium- and large sized mammals at Neumark, small animals such as turtles may simply have been attractive because of their specific taste, or because of other non-nutritional characteristics. In historic times, the terrapin was also appreciated for its medicinal values^19^, as stated in an early 19th century encyclopedia entry by the zoologist A. G. Desmarest:

“There are yet living European pond turtles to be found at sundry apothecaries in Paris, who do bring them from Provence to prepare broths, these being held in high esteem for afflictions of the lungs, and for restoring strength wasted through the excesses of amorous indulgence. They are consumed in the regions where they are taken, yet their flesh, so far as I have been able to judge, is far inferior in quality to that of the American turtles.”^38^, p. 262].

While an interesting historical observation (see also^19^), medicinal usage holds limited relevance here in view of its invisibility in the archaeological record. Given the archaeological context of the Neumark-Nord *Emys* material, with its abundant evidence for intensive exploitation of hundreds of large mammals, food provisioning by children^39^, capturing terrapins when encountered around the lakes may have been a factor here. It is also possible that collection was motivated by the (small) carapaces, which, according to the ethnographic record - as well as some archaeological data^40^-, can be used as containers. The processing evidenced by cut mark locations, particularly the careful cleaning of carapace elements from both soft tissues and skeletal remains, suggests preparation for such use. The organic keratin covering (scutes) would not preserve archaeologically, precluding assessment of potential applications. The presence of two carapaces within the Neumark-Nord 2/2B “grease rendering” concentration^10^ does seem to suggest a “scoop-like” use, though this must also remain a speculative interpretation.

What is clear, is that these Neumark-Nord data constitute the first evidence of turtle exploitation by Neanderthals north of the European mountain chains, beyond the Mediterranean basin. These small animals were collected within a landscape inhabited by a wide range of medium-sized to very large mammals, all of which were exploited by Neanderthals during the Last Interglacial^10,20,25^. This spectrum includes both the ~ 1 kg pond terrapins discussed here as well as adult male straight-tusked elephants, which could weigh over 10,000 times more.

This high-resolution evidence for a broad Neanderthal game spectrum - from small terrapins to the largest land mammals of the Pleistocene – is accompanied by strong indications of extensive mammal carcass exploitation for a range of nutrients, including bone grease^10^. Prey processing activities were spatially distributed across the landscape: large ungulates killed and initially processed at one location, with some skeletal parts - possibly first cached for future use – transported to a central location for grease rendering^10^. This well-documented fragmentation of the prey processing sequence highlights the necessity of studying Neanderthal subsistence at the landscape scale.

Besides the archaeologically very visible focus on animal resources, subsistence activities around these water bodies would have included plant food collection, providing an important part of the diet of these Last Interglacial foragers^10^. The Neumark-Nord 2/2B excavations yielded charred remains of hazelnut (*Corylus avellana*), acorn (*Quercus* sp.) and blackthorn (or: sloe plum, *Prunus spinosa*)^41^, very probably reflecting such food items. Many more potential food plants are amongst the macro-botanically identified remains of more than 190 plant species^42^ and in the pollen assemblage recovered from the two Neumark-Nord basins^24^^,43^. These include carbohydrate sources such as cattail (*Typha* sp.) and reed (*Phragmites australis*), as well as berries and other fruits alongside potential green vegetables or plants usable for both their greens and seeds (see^10^). The strikingly – possibly: anthropogenic - open^28^ areas around the basins, with an abundance of *Corylus*^24,28^, would have afforded easy access to plant food, including grass seeds, well-documented as a component of the Neanderthal diet^8^.

In conclusion, the abundance of medium- and large-sized mammal fossils found alongside the *Emys* remains described here does not support the hypothesis that depletion of large game, i.e. resource scarcity, compelled Neanderthals to repeatedly exploit smaller prey. Within the context of the Neumark-Nord Last Interglacial landscape, we suggest that other variables than macronutrients per se played a role in the repeated harvesting of pond terrapins from these water bodies. Whether this entailed specific taste preferences, children’s activities and/or the production of “scoops” for the Neanderthal cuisine or other reasons, remains unclear. The evidence does offer a compelling illustration of the breadth of Neanderthal prey selection within a – in Pleistocene terms: very - brief time span and a geographically confined lakeside setting—one in which Neanderthal presence left a discernible impact on the local environment^28^.

## Materials and methods

Based on studies by Karl^32^ and Böhme^31^, identification and attribution of carapace and plastron remains of the European pond turtle follows criteria and nomenclature presented by Ullrich^44^. Cut marks were identified by SGW and LK with a hand held lens (10-40x magnification) and DinoLite PRO portable microscope (−200x magnification). For documentation and visualization, high resolution 3D-scans (LIM ToolScan R360) in the zooarchaeological and taphonomical collection at MONREPOS and the Institute of Forensic Technology of the Landeskriminalamt Nordrhein-Westfalen were used. For validation the cut marks were compared to traces caused by biotic and abiotic agents identified using the zooarchaeological and taphonomic collection of the Archaeological Research Centre and Museum for Human Behavioural Evolution, MONREPOS, as well as diagnostic criteria published by Fernández-Jalvo and Andrews^45^.

Estimates of carapace size have been provided by Böhme^31^ for the material from NN1. They are calculated from measurements of the length of half the hyo-/hypoplastron suture. Based on his data the conversion factor to obtain overall carapace length is 3,49.

## Data Availability

All data needed to evaluate the conclusions in the paper are present in the paper and the Supplementary Materials. All information, documentation, and materials from Neumark-Nord are the property of the State of Saxony-Anhalt, with the State Heritage Office of Saxony-Anhalt [Landesamt für Denkmalpflege und Archäologie, Sachsen-Anhalt (LDA-LSA)] in Halle, Germany, serving as the responsible administrative authority. The material remains part of several ongoing research projects led and organized by SGW, LK, WR and the LDA-LSA.
